# Editorial: Comparative Genetics of NK Cell Receptor Families in Relation to MHC Class I Ligands and Their Function

**DOI:** 10.3389/fimmu.2020.00561

**Published:** 2020-04-01

**Authors:** Lutz Walter, Ronald Bontrop

**Affiliations:** ^1^Primate Genetics Laboratory, German Primate Center, Leibniz Institute for Primate Research, Göttingen, Germany; ^2^Comparative Genetics and Refinement, Biomedical Primate Research Centre, Rijswijk, Netherlands

**Keywords:** NK receptors, MHC, genomics, NHP, animal model, livestock

Natural killer (NK) cells are an essential part of the innate immune system to fight against infections of viral, bacterial, fungal, or parasite origin and have been implicated in eliminating cancerous cells. The NK cells' killing activity have a direct (infected cells) or indirect (other immune cells) effect on immunity. In addition, NK cells have an important immune regulatory role by production of cytokines and chemokines, for instance, in reproduction. The activity of NK cells is controlled by various receptors belonging to the immunoglobulin-like and the C-type lectin-like protein families. These receptors mediate either inhibitory or stimulatory signaling and the integration of their signals essentially determines the activity of NK cells. Important NK cell receptors are the family of killer cell immunoglobulin-like receptors (KIR) and the killer cell lectin-like receptor families of Ly49 and CD94/NKG2 proteins. The ligands are not known for all receptors, but many members bind MHC class I or MHC class I-like proteins. The enormous and unprecedented variability of MHC class I allotypes is well-known and some NK cell receptors turned out to be highly variable (e.g., KIR) as well, whereas others are conserved such as CD94/NKG2A or NKG2D. Not surprisingly, combinations of receptor and ligand alleles were found to be associated with susceptibility and resistance to a variety of human diseases, mostly immune diseases (1). Such associations are not so well recorded in most animal models, but were reported in an important non-human primate model of AIDS (2, 3), indicating that genetic variability of NK cell receptors and ligands impacts animal models of human diseases as well as livestock and calls for thorough analyses. Hence, this Research Topic addresses comparative genetics of NK cell receptor families and MHC class I ligands and their function.

Chickens were previously reported to possess numerous *KIR*-like (*ChIR*) genes in their genome. In their original article, Meziane et al. report on the complexity of chicken *ChIR* haplotypes. The data suggest that *ChIR* haplotypes have relatively stable number of genes and low number of gene copy number variations.

Schwartz et al. describe in an original article the *KIR* genomic organization in goat and compare these data to sheep and cattle. Goats and sheep *KIR* are characterized by an own *KIR* subgroup and by absence of typical three-domain *KIR* genes.

By comparing *KIR3DL3* genes in primates, Leaton et al. report in an original article that polymorphisms of the enigmatic KIR3DL3 receptor are not focused on ligand-binding sites known from other KIRs, but are instead found in the D1 domain at a site thought to involve KIR-KIR interaction.

In their original article, Bruijnesteijn et al. studied the extent and potential functional consequences of alternative splicing of human and rhesus macaque *KIR* genes. The study illustrates that KIR's may come in many isoforms. Further, they observed convergent evolution of the rhesus macaque framework *KIR* gene *KIR3DL20* and the non-conserved human *KIR2DL5* gene.

Wroblewski et al. review current knowledge of *KIR* and *MHC class I* genes in hominid (humans and great apes) primates. The authors point out the intimately linked co-evolution of both gene families and use the power of comparative genetics/genomics to explain the current set and variability of human *KIR* and *HLA class I* genes and ensuing functional consequences.

Typing of NK cell receptor genes in non-human species often profits from technical developments achieved in humans. In an original article, Wagner et al. present the development of sophisticated human *KIR* gene typing based on high-throughput sequencing. Typing of 300,000 samples resulted in identification of 2,000 previously unreported alleles of human *KIR* genes.

In an original article, Augusto et al.. describe the usage of single nucleotide polymorphisms of the human *KIR* genomic region as markers for presence and absence of individual *KIR* genes and determination of *KIR* haplotypes. Differences in various human populations from different geographic regions are shown.

Mahaweni et al. review the effects of HLA class I proteins expression on the cell surface of multiple myeloma cells. They discuss how these target cells can maintain a sufficiently high level of HLA-E expression to prevent recognition and activation of NK cells.

Comparative analyses of genes and genomic regions reveal insights into the rapid evolution of genes encoding NK cell receptor and their MHC class I ligands. This not only provides valuable clues to understand the current set of genes and alleles in our species and of human-specific traits in immune responses and diseases, such studies are highly valuable to better understand and refine animal models of such diseases or animals that are of immense economic value. Although the collection of articles in this Research Topic adds to our understanding, we feel that there is still some way to go and to include further important species for more detailed and comprehensive insights. High-throughput sequencing at affordable costs unquestionably helps to achieve this.

## Author Contributions

LW and RB wrote the manuscript.

### Conflict of Interest

The authors declare that the research was conducted in the absence of any commercial or financial relationships that could be construed as a potential conflict of interest.
